# Unusual clinical manifestation and challenging serological interpretation of syphilis: insights from a case report

**DOI:** 10.1186/s12879-021-06199-0

**Published:** 2021-06-02

**Authors:** F. Magri, M. G. Donà, C. Panetta, M. Pontone, F. Pimpinelli, N. Cameli, A. Cristaudo, M. Zaccarelli, A. Latini

**Affiliations:** 1 Department of Dermatology, Policlinico Umberto I, Sapienza University of Rome, Rome, Italy; 2 STI/HIV Unit, San Gallicano Dermatological Institute IRCCS, Rome, Italy; 3 Laboratory of Dermatopathology, San Gallicano Dermatological Institute IRCCS, Rome, Italy; 4 Clinical Pathology and Microbiology, San Gallicano Dermatologic Institute IRCCS, Rome, Italy; 5 Department of Dermatology, San Gallicano Dermatological Institute IRCCS, Rome, Italy; 6 Clinical Department, National Institute for the Infectious Diseases Lazzaro Spallanzani, Rome, Italy

**Keywords:** HIV, Syphilis, Serological test, Case report

## Abstract

**Background:**

The clinical manifestations of recent syphilis can be variable, with typical and atypical patterns. Several conditions may cause atypical clinical aspects, including human immunodeficiency virus (HIV) co-infection. Besides the clinical features, co-infections may completely alter syphilis serological tests, causing interpretative difficulties and diagnostic delays. Aim of the work is to describe the difficulties encountered during the diagnostic evaluation of atypical skin manifestations and of the serology for syphilis of an HIV-infected patient who had contracted it several times.

**Case presentation:**

In 2020, a 52-year old HIV-positive bisexual male patient was admitted to our department with a 4-month history of moderately itchy cutaneous lesions localized at his neck, trunk and arms. In 2013, the patient presented with a classic syphilitic roseola of the trunk and a secondary syphilis was diagnosed, with increased levels of rapid plasma reagin (RPR), Treponema pallidum hemagglutination assay (TPHA), anti-Treponema pallidum IgM and IgG Index. A second episode occurred in 2018, as a primary syphilis with multiple ulcerative lesions of the penis, and increased levels of RPR, IgG and IgM. In 2019, a further episode of secondary syphilis was treated with Doxycycline. In 2020, erythematous and papular lesions with vesicular components and urticarial erythema multiforme (EM)-like lesions were present at the neck, trunk and arms. Serological tests and Nucleic Acid Amplification Test (NAAT) for Treponema Pallidum were performed, as well as a cutaneous biopsy with histological and immunohistochemical evaluation of one lesion. NAAT was negative for *T. pallidum*. Serological test results were discordant with a new syphilis infection, showing only increased levels of RPR and anti-Treponema IgG. The cutaneous biopsy revealed a non specific histological pattern, while the immunohistochemical evaluation with anti-spirochetal antibodies was mandatory for the diagnosis of recent syphilis, showing clusters of rod-shaped elements, some of which with spiral form, focally present at the epidermis and adnexal structures.

**Conclusions:**

Nowadays, syphilis may present with atypical clinical and serological features. Physicians should be aware of these possible alterations and consider syphilis even in case of uncommon clinical aspect and unclear serological tests. Cutaneous biopsy and immunohistochemical exam may be mandatory for the diagnosis.

## Background

Syphilis is considered as a “great imitator” because of its wide clinical variability. The clinical manifestations of recent syphilis are variable.

Several cutaneous lesions may be observed, such as macular, papular, lichenoid and pustular lesions, and, for this reason, syphilis recognition is frequently challenging, even for expert dermatologists.

Furthermore, several conditions may contribute to atypical clinical manifestations, including hepatitis or human immunodeficiency virus (HIV) co-infections as well as recreational drug use. In some of these situations, the diagnosis of syphilis may be difficult with consequent therapeutic delay.

Serological tests with the association of treponemal and nontreponemal exams are essential to achieve the correct diagnosis of syphilis, especially in case of atypical clinical presentation [1]. However, they may be completely altered or discordant with the stage of infection, especially in case of multiple re-infections, causing interpretative difficulties and diagnostic delay.

Here, we present a case of secondary syphilis, with atypical urticarial and erythema multiforme (EM)-like aspect and discordant serology results, in a 52 year-old patient with HIV and previous syphilis episodes.

## Case presentation

**Fig. 1:**
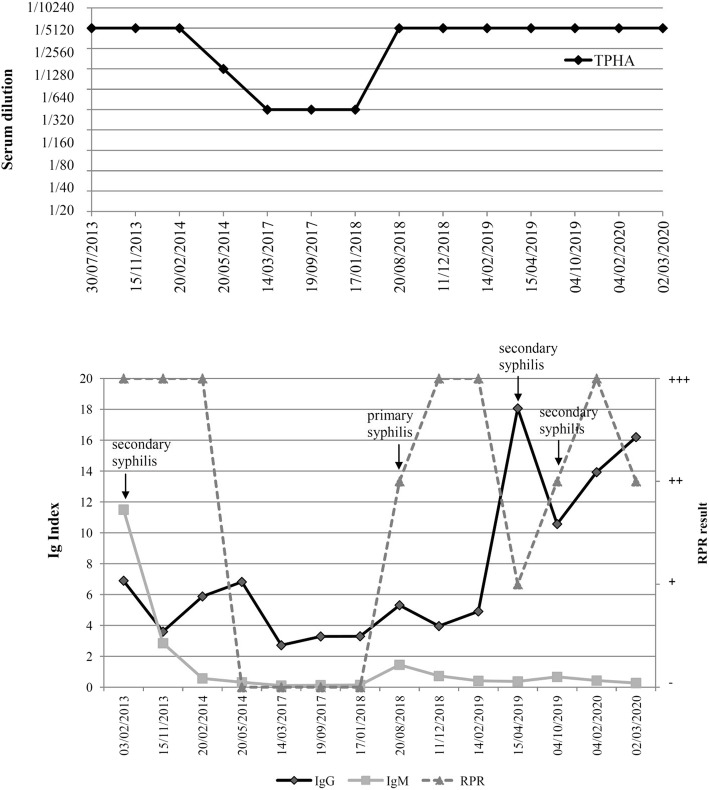
**a)** Results of the Treponema pallidum hemagglutination assay (TPHA) from the first to the most recent syphilis episode. **b)** Results of the treponemal serological tests for IgG and IgM, as well as for the Rapid Plasma Reagin (RPR) during the entire clinical history since the first syphilis diagnosis. The single episodes are highlighted

In February 2020, a 52-year old HIV positive bisexual male patient was admitted to our Department with a 4-month history of moderately itchy cutaneous lesions localized at his neck, trunk and arms. The patient had been under antiretroviral therapy for 13 years. At that moment, the patient was under treatment with emtricitabine/rilpivirine/tenofovir alafenamide (Odefsey®), with successful results. In fact, the patient had been virally suppressed for 10 years, with no diagnosis of AIDS nor AIDS-defining diseases. At the moment of our observation, CD4 count was 622 cells per mm^3^, CD8 count was 771 cells per mm^3^, while CD4/CD8 ratio was 0.76. Personal medical history was negative for hepatitis B or C virus co-infections, while it was positive for previous hepatitis A virus. Furthermore, his medical history was positive for relapsing cutaneous and mucosal herpes simplex virus infections and for three previous episodes of syphilis, in 2013, 2018 and 2019. First syphilis diagnosis dated back to 2013, as a secondary syphilis. Clinically, the patient presented with a classic syphilitic roseola of the trunk. Serological tests showed increased levels of rapid plasma regain (RPR), Treponema pallidum hemagglutination assay (TPHA), anti-Treponema pallidum IgM and IgG Index, as reported in Fig. 1. The patient refused injection therapy and underwent a systemic treatment with oral doxycycline 100 mg twice a day for 20 days, with resolution of the lesions in few days and negativity of RPR and IgM in 10 months.

The second episode of syphilis occurred in August 2018, as a primary syphilis with multiple ulcerative lesions of the penis, and serological tests showing increased levels of RPR, IgG and IgM. Doxycycline 100 mg twice a day for 20 days was prescribed, with normalization of IgM and RPR after 4 months.

The third episode of syphilis took place in April 2019, as a secondary syphilis, and was treated again with Doxycycline.

**Fig. 2:**
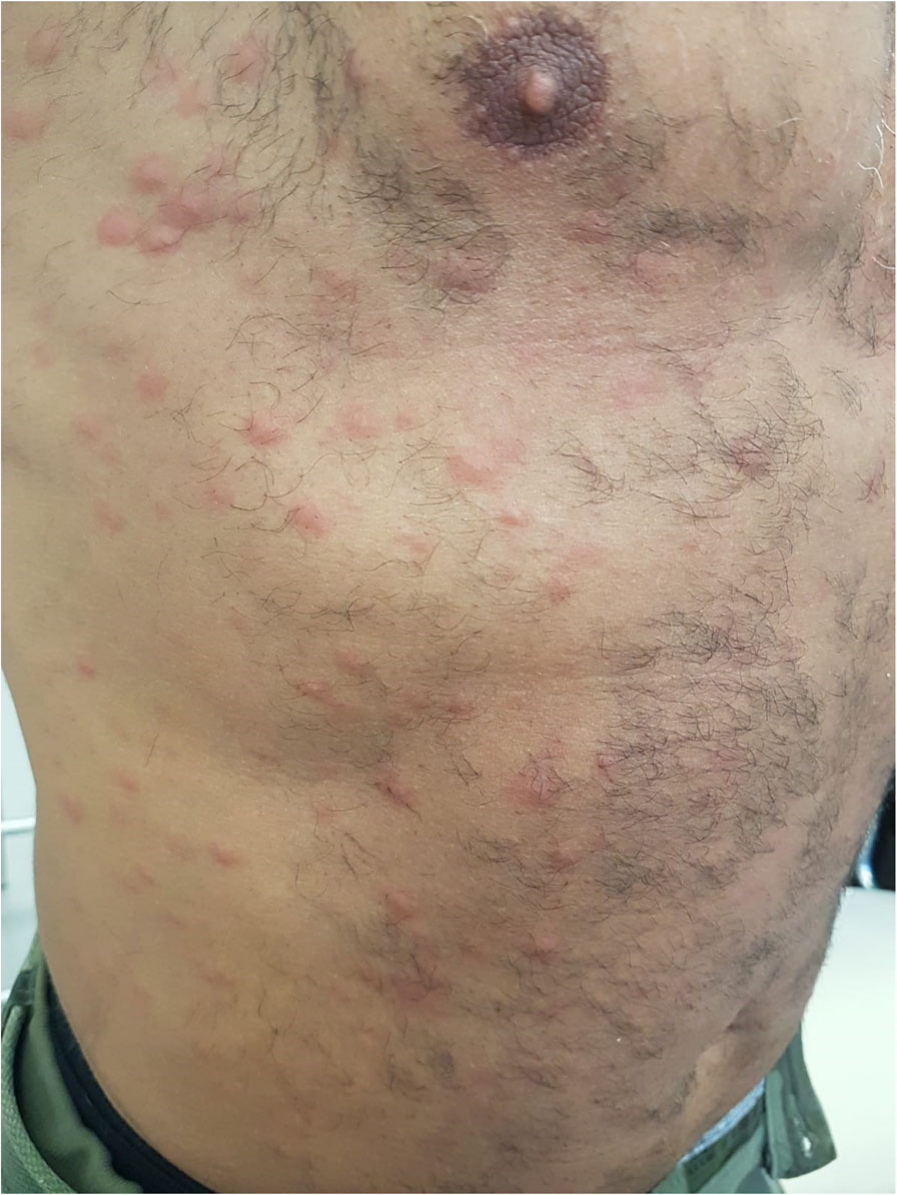
Diffuse erythematous and urticarial lesions of the trunk, with erythema multiforme-like components; similar lesions were present also at the neck and arms

**Fig. 3:**
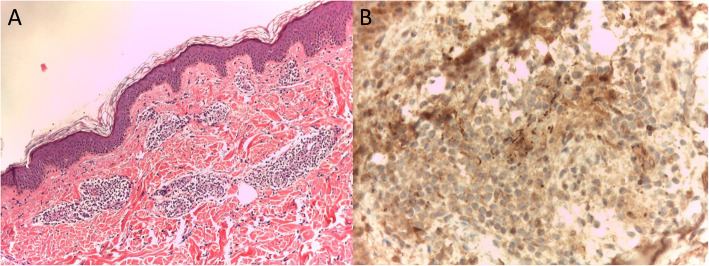
**a)** Histological examination showing mild hyperkeratosis and acanthosis of the epidermis; at the superficial and middle dermis, a non-specific parvocellular inflammatory infiltrate of lymphocytes, plasm cells and rare histiocytes with predominant perivasal distribution was present; dermal vessels showed thickened cell wall and endothelial cell swelling, with no clear signs of vasculitis (40x magnification); **b)** Immunohistochemical staining with anti-spirochetal antibodies, showing clusters of rod-shaped elements, some of which with spiral form, focally present at the epidermis and adnexal structures (100x magnification)

At the moment of our visit in February 2020, on physical examination, erythematous and papular lesions with vesicular components and urticarial erythema multiforme (EM)-like lesions were present at the neck, trunk and arms (Fig. 2). The patient reported a stable partner and no occasional intercourses in the last year. Serological syphilis tests were discordant with active infection. In fact, increased levels of RPR and IgG were present, but anti-Treponema IgM were negative. Nucleic acid amplification test (NAAT) for syphilis performed on an erythematous-papular scapular lesion was negative for Treponema pallidum. Based on the polimorfo-like aspect of the lesions, a topical and systemic steroid and antihistamine treatment was started, with no benefit. In consideration of the resistance to local treatments, an incisional biopsy with histological exam of a cutaneous lesion of the trunk was performed. The histological exam revealed the presence of mild hyperkeratosis and acanthosis of the epidermis; at the superficial and middle dermis, a non-specific parvocellular inflammatory infiltrate with predominant perivasal distribution was also present. The dermal infiltrate was mainly composed by lymphocytes, plasm cells and rare histiocytes; dermal vessels showed thickened cell wall and endothelial cell swelling, with no clear signs of vasculitis (Fig. 3a). Immunohistochemical analysis with anti-spirochetal antibodies was mandatory for the diagnosis, showing clusters of rod-shaped elements, some of which with spiral form, focally present at the epidermis and adnexal structures (Fig. 3b).

Indeed, a diagnosis of secondary syphilis was made, even if serological syphilis tests were discordant with active infection. A single intramuscular dose of 2.400.000 IU of penicillin in association with intramuscular betamethasone 4 mg was administered, once a week for 3 weeks with resolution of the clinical condition and no relapses at a 3-month follow up.

## Discussion and conclusions

After a long period of declining incidence, syphilis is currently a reemerging disease worldwide, especially between subjects at increased risk of sexually transmitted diseases (STD). Several studies have observed that a high seroprevalence of syphilis is present in HIV-infected patients, in particular among men who have sex with men (MSM) [2]. A review calculated a 9.5% prevalence of syphilis between HIV-infected subjects [3]. The co-occurrence of these diseases is extremely important; syphilis may influence the HIV course inducing a reduction of CD4 cells and an increase in HIV viral load; additionally, syphilis may increase the risk of HIV transmission [4].

Besides the epidemiological aspect, the co-existence of these two infectious diseases is of interest also for clinical and diagnostic features. Firstly, HIV-positive patients show more commonly atypical clinical aspects of syphilis [5]. In fact, in our patient, recent syphilis did not appear with the classic syphilitic roseola of secondary infection, but with an uncommon urticarial and EM-like cutaneous eruption. Only eleven cases of EM-like syphilis have been reported in the literature worldwide [6].

Furthermore, particularly in HIV-infected patients, syphilis serological tests may not correlate with the clinical presentation and consequently are not helpful in achieving the correct diagnosis.

Centers for Disease Control and Prevention (CDC) guidelines highlight that the interpretation of nontreponemal and treponemal tests in HIV-infected patients is essentially the same of the general population. However, altered serological tests are possible among HIV-positive subjects with syphilis: post-therapy serological values may be higher than expected (high serofast) or fluctuating; false-negative serological tests or delayed seroreactivity have been also described [7, 8].

In our patient, serological tests were unclear and NAAT was negative during the last episode of syphilis. Indeed, the diagnosis was made with the help of a histological exam and immunohistochemical staining. Our case was substantially in accordance with the recent considerations by Ghanem et al. The authors highlight that a negative NAAT does not rule out syphilis, and that immunohistopathology is the preferred method when tissue sections are available [1].

At the last syphilis episode, anti-Treponema IgM index was negative during the active infection, while an increase of RPR and IgG was evident. Probably, these alterations were a consequence of abnormal B cell activation and abnormal immune regulation present in HIV patients, even if under successful antiretroviral treatment. In our patient, CD4 count was > 350 cells per mm3 in all his HIV history, suggesting that syphilis recurrence was not fully related to immunosuppression.

RPR and IgG index values were particularly useful for the interpretation of acute episodes, as reported in Fig. 1; indeed, these parameters could be considered when evaluating syphilis tests in HIV-infected subjects. IgG and RPR were the only parameters showing an increase in all four early syphilis episodes of the patient. Instead, increased levels of IgM were only present during the first episode. TPHA evaluation was significant only during the first episode. Then, during subsequent reinfections, TPHA levels were rather fluctuating and for this reason not useful for patient monitoring.

In our case, first three occurrences of syphilis were easily suspected, in consideration of their classic clinical aspect. In the last episode, which presented with an atypical cutaneous eruption, discordant serological tests and negative NAAT, the cutaneous biopsy with immunohistochemical evaluation was necessary.

In conclusion, we presented this clinical case to highlight that HIV-infected patients with coexisting syphilis may present atypical clinical and serological features. Focal points to emphasize are: TPHA is not an essential parameter in syphilis monitoring; cutaneous biopsy is mandatory in case of atypical clinical aspect with associated epidemiological suspect; immunohistochemical evaluation is essential in case of non specific histological patterns; PCR negativity from non erosed cutaneous lesions cannot rule out an active infection. Physicians must be aware of these possible alterations and must always consider a possible syphilis episode even in case of uncommon clinical aspect and unclear serological tests.

## Data Availability

Not applicable.

## Abbreviations

CDC: Centre for Disease Control and Prevention
EM-like: Erytema multiforme-like
HIV: Human Immunodeficiency Virus
MSM: Men who have sex with men
NAAT: Nucleic Acid Amplification Test
RPR: Rapid plasma reagin
STD: Sexually transmitted disease
TPHA: Treponema pallidum hemoagglutination assay
